# Immunotherapy for advanced lung cancer combined with surgery for mediastinal myxofibrosarcoma: a case report

**DOI:** 10.1186/s40792-019-0596-7

**Published:** 2019-02-26

**Authors:** Tetsuya Fukui, Yusuke Wakatsuki, Tadashi Matsukura

**Affiliations:** Department of General Thoracic Surgery, Japanese Red Cross Fukui Hospital, 2-4-1 Tsukimi Fukui, Fukui, 918-8501 Japan

**Keywords:** Mediastinal myxofibrosarcoma, Surgical resection, Lung cancer, Immunotherapy

## Abstract

**Background:**

It is unclear whether simultaneous primary neoplasm resection and immunotherapy for advanced lung cancer is safe. We report a case of an elderly man with advanced lung cancer and myxofibrosarcoma.

**Case presentation:**

The advanced lung cancer was treated with pembrolizumab, and partial response was achieved in 3 months. However, the mediastinal cyst enlarged rapidly. We resected the mediastinal tumor and diagnosed it as myxofibrosarcoma. The postoperative course was uneventful. Immunotherapy was resumed after the operation without any adverse effects. No recurrence of mediastinal sarcoma or progression of lung cancer was found until the patient died in an accident 8 months after surgery.

**Conclusion:**

Surgery for mediastinal sarcoma could be performed safely in combination with immunotherapy for advanced lung cancer.

## Background

Primary mediastinal myxofibrosarcomas are rare, and surgical resection is the main treatment. Immune checkpoint inhibitors can have great efficacy in some advanced non-small cell lung cancers (NSCLCs), but cause unique immune-related adverse effects [1]. It is unclear whether simultaneous primary neoplasm resection combined with immunotherapy is safe. This is the first report on the combination of immunotherapy for advanced NSCLC and surgery for mediastinal sarcoma.

## Case presentation

**Fig. 1:**
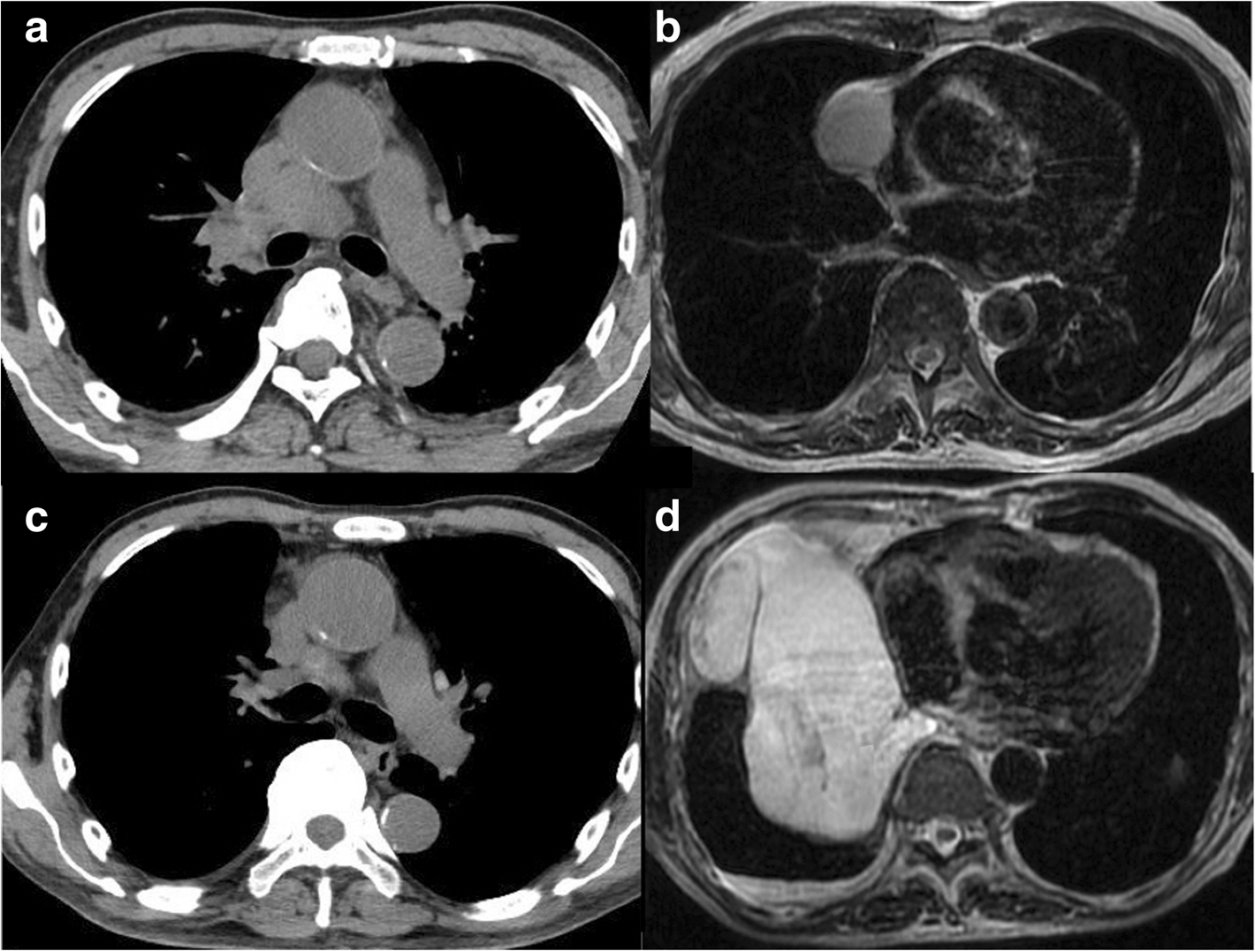
Computed tomography revealed hilar and mediastinal lymph node enlargement (**a**). Magnetic resonance imaging demonstrated a high-intensity anterior mediastinal tumor on T2-weighted images (**b**). Treatment with pembrolizumab yielded a partial response from the non-small cell lung cancer (NSCLC) (**c**) but the mediastinal cyst enlarged extremely rapidly within 3 months (**d**)

**Fig. 2:**
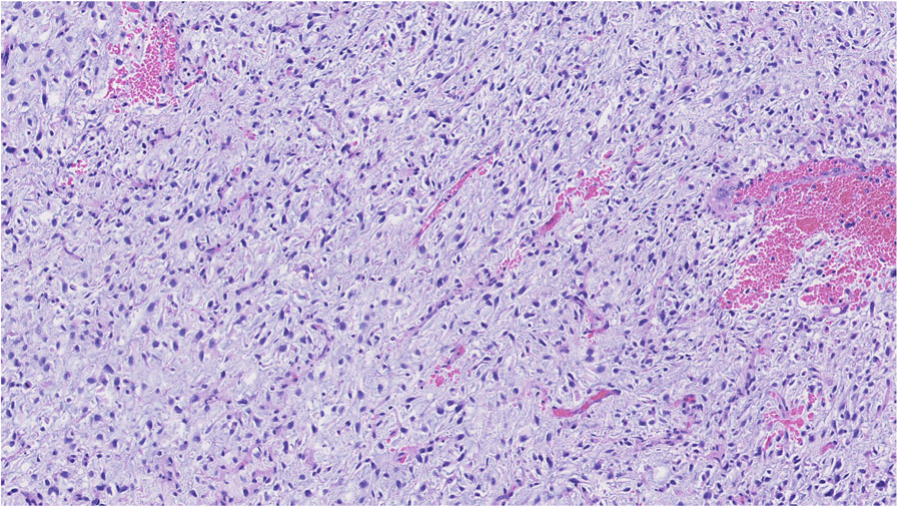
Intermediate power magnification of × 150 reveals spindle cells in a rich myxoid stroma (HE staining)

An 81-year-old man was referred to our center due to an abnormal shadow finding on a chest plain radiograph with chief complaints of cough and hip pain. The patient had no smoking history. His Eastern Cooperative Oncology Group performance status was 1. He had chronic renal failure; hence, we did not use any contrast agents for computed tomography (CT) or magnetic resonance imaging (MRI). CT revealed a lung nodule in the right upper lobe, mediastinal lymph node enlargement (Fig. 1a), an osteolytic lesion of the left iliac bone, and an anterior mediastinal cyst measuring 5 cm in size. The former three lesions showed significant fluorodeoxyglucose (FDG) accumulation on FDG-positron emission tomography (PET), and advanced lung cancer with clinical stage T1bN2M1b was suspected. Endobronchial ultrasound-guided transbronchial needle aspiration indicated that the lung adenocarcinoma was ALK*-*negative, with wild-type EGFR and a high PD-L1 tumor proportion score (95%). The latter mediastinal cyst showed no abnormal accumulation on PET-CT, and MRI demonstrated high intensity on T2-weighted images (Fig. 1b). Magnetic resonance cholangiopancreatography performed 1 year before the appearance of the mediastinal tumor indicated that the tumor occurred within the year. We determined that the patient had double neoplasms with advanced NSCLC and a mediastinal tumor. It was unknown whether the mediastinal tumor was benign or malignant. The patient’s prognosis seemed to depend on the treatment of the NSCLC rather than the mediastinal tumor. Therefore, we prioritized lung cancer treatment. Blood screening before immune checkpoint inhibitor (ICI) therapy (such as autoimmune and endocrine function, including thyroid function) showed normal data with the exception of renal failure. Radiation therapy with 39 Gy to the left iliac bone was performed, and pembrolizumab was administered as the first-line treatment; we observed the mediastinal tumor. Treatment with pembrolizumab yielded a partial response from the NSCLC in 3 months (Fig. 1c), but the mediastinal cyst enlarged extremely rapidly to 15 cm in diameter (Fig. 1d) and was suspected to be a sarcoma. Owing to the NSCLC treatment, the size of the mediastinal tumor rather than the NSCLC seemed to be more directly associated with prognosis. We decided to resect the mediastinal tumor under immunotherapy. Two weeks after the fourth administration of pembrolizumab, we performed mediastinal tumor excision by posterolateral thoracotomy, combined with resection of the right phrenic nerve and pericardium, which was reconstructed with an expanded polytetrafluoroethylene patch. We achieved a pathologically curative resection, but the tumor tended to infiltrate the surrounding structures and bled easily even with careful dissection. The blood loss was 1400 ml, and the patient received blood transfusion. The tumor was filled with a jelly-like substance and weighed 920 g. Pathological findings revealed spindle cells in a rich myxoid stroma with a high rate of mitosis and multinucleated pleomorphic nuclei (Fig. 2). Immunohistochemical staining was negative for CD68, bcl2, ckit, ALK, SMA, desmin, S100, AE1/3, EMA, STAT6, and MUC4 and positive for CD99, CD34, MDM2, and CDK4. The pathological diagnosis was myxofibrosarcoma with no expression of PD-L1 (tumor proportion score, 0%). The postoperative course was uneventful. Immunotherapy was resumed 3 weeks after the operation, and a total of 13 courses were administered without any adverse effects. No adverse events were noted on the blood screens performed. No recurrence of mediastinal sarcoma or progression of NSCLC was found until the patient died in an accident 8 months after surgery. Patient consent was obtained for publication of this case report.

## Discussion

This is the first reported case of a combination of immunotherapy for lung cancer and surgery for mediastinal sarcoma. The present case highlighted two important clinical issues.

First, mediastinal sarcoma resection can be performed safely in combination with immunotherapy. Pulmonary resections for lung cancer after ICI therapy have been reported to be feasible [2]. Neoadjuvant nivolumab therapy had an acceptable side-effect profile and was not associated with a delay in lung cancer surgeries with a median interval between administration of the last (second) dose and surgery of 18 days (range, 11 to 29) [3]. ICIs can demonstrate great efficacy in some advanced NSCLCs but cause unique immune-related adverse effects [1]. We need to be watchful for ICI-related pneumonitis, particularly in thoracic surgery. The incidence of pneumonitis is reported to be approximately 5% and the time-to-onset ranges from 9 days to 19.2 months. [4]. The optimal timing of the operation following pembrolizumab administration is unclear. However, an interval of at least 10 days after the first dose may be preferable to check for the presence of pneumonitis. We should preserve the lung as much as possible and carefully check for pneumonitis perioperatively. In this elderly patient with chronic renal failure and advanced NSCLC, surgery for a giant mediastinal sarcoma using an expanded polytetrafluoroethylene patch combined with immunotherapy was performed without any adverse events. After the surgery, pembrolizumab for the NSCLC was resumed and good efficacy was observed without any adverse effects. Accumulation of further cases is needed to definitively determine the optimal strategy and protocol for managing patients undergoing surgery combined with immunotherapy.

Second, in the case of multiple primary neoplasms, the treatment for one neoplasm, including immunotherapy, may influence the progression of the other tumors. In the present case, myxofibrosarcoma occurred before immunotherapy, but progressed extremely rapidly during the first 3 months of treatment. We cannot confirm if this was due to natural tumor progression of the myxofibrosarcoma or if it was affected by immunotherapy. The recurrence rate of myxofibrosarcoma in the extremities is reported to be 16.8% and is definitely higher than those of all other soft tissue sarcomas, which are reported to have a recurrence rate of 10% [5]. Primary mediastinal myxofibrosarcomas are rare, and only four cases have been reported to date [6, 7]. A case of mediastinal myxofibrosarcoma in which local recurrences were detected after three reoperations was reported [6]. Mediastinal myxofibrosarcoma may be a progressive disease, but it remains possible that it was affected by the NSCLC treatment or immunotherapy.

## Conclusion

Surgery for mediastinal sarcoma can be safely performed combined with immunotherapy for advanced NSCLC. In the case of multiple primary neoplasms, treating one neoplasm using immunotherapy may affect the progression of the other tumors. While immunotherapy has recently been widely adopted, further studies are needed to assess its effects on multiple primary neoplasms. These studies would help in deciding the course of treatment for similar cases in the future.

## Abbreviations

CT: Computed tomography
FDG: Fluorodeoxyglucose
ICI: Immune checkpoint inhibitor
MRI: Magnetic resonance imaging
NSCLC: Non-small cell lung cancer
PET: Positron emission tomography
